# GaAs epilayers grown on patterned (001) silicon substrates via suspended Ge layers

**DOI:** 10.1038/s41598-019-53949-x

**Published:** 2019-11-26

**Authors:** Andrea Ballabio, Sergio Bietti, Andrea Scaccabarozzi, Luca Esposito, Stefano Vichi, Alexey Fedorov, Anna Vinattieri, Cosimo Mannucci, Francesco Biccari, Akos Nemcsis, Lajos Toth, Leo Miglio, Massimo Gurioli, Giovanni Isella, Stefano Sanguinetti

**Affiliations:** 1 L-NESS, Physics Department, Politecnico di Milano, via Anzani 42, 22100 Como, Italy; 2 L-NESS, Material Science Department, Università degli Studi di Milano-Bicocca, via Anzani 42, 22100 Como, Italy; 3 L-NESS, CNR-INFM, via Anzani 42, 22100 Como, Italy; 4 Physics and Astronomy Department, Università degli Studi di Firenze, via G. Sansone 1, 50019 Sesto Fiorentino, Italy; 5 European Laboratory for Non-linear Spectroscopy (LENS), University of Florence, Via N. Carrara 1, 50019 Sesto Fiorentino, Italy; 6 Institute of Microelectronics and Technology, Obuda University, Tavaszmezo u. 17, 1084 Budapest, Hungary; 7 Institute for Technical Physics and Materials Science MTA-EK, Konkoly-Thege u. 29, 1121 Budapest, Hungary

**Keywords:** Semiconductors, Surfaces, interfaces and thin films, Design, synthesis and processing, Surface patterning, Materials science, Optoelectronic devices and components

## Abstract

We demonstrate the growth of low density anti-phase boundaries, crack-free GaAs epilayers, by Molecular Beam Epitaxy on silicon (001) substrates. The method relies on the deposition of thick GaAs on a suspended Ge buffer realized on top of deeply patterned Si substrates by means of a three-temperature procedure for the growth. This approach allows to suppress, at the same time, both threading dislocations and thermal strain in the epilayer and to remove anti-phase boundaries even in absence of substrate tilt. Photoluminescence measurements show the good uniformity and the high optical quality of AlGaAs/GaAs quantum well structures realized on top of such GaAs layer.

## Introduction

III–V semiconductors, in particular As-based compounds, are fundamental for a number of essential opto-electronic and photonic applications. GaAs is probably one of the most studied III–V semiconductors as it has demonstrated abilities for high-speed and high power applications^1,2^, laser diodes and RF devices for optical network and communications thanks to high mobility and direct bandgap properties^3,4^. A fundamental step ahead would be the monolithic integration of these photonic components on silicon chips, coupling the standard Si-based micro-electronic technology with the high quality opto-electronic properties of the III–V semiconductors^5^. For a monolithic integration, direct epitaxy of GaAs on Si(001) is required^6,7^.

However, the heteroepitaxial growth of compound semiconductors on silicon is still challenging despite several decades of research^6,7^. The first challenge is how to control the density of dislocations, which arise from the lattice misfit between the Si substrate and the epilayer (for GaAs/Si this difference amounts to ≈4.2%). Dislocations are required to relax the misfit strain but often negatively affect the optical and electrical properties of active layers. In particular, threading dislocations (TDs), reaching the surface, drastically decrease carrier lifetime and eventually reduce the life span of opto-electronic devices^8^. Thanks to the strong similarity between GaAs and Ge lattice parameters and the large number of effective strategies developed to obtain high quality Ge epilayers on Si, a Ge buffer layer is usually inserted to solve the lattice mismatch issue^9–15^. The second concern is due to the difference between the thermal expansion coefficients (for GaAs/Si it is ≈123%) which causes wafer bowing and cracks formation in the epilayer. This becomes especially relevant for film thicknesses beyond a few micrometers^6,7,16–19^.

An effective strategy to overcome both these limits is the use of 3D growth of thick III–V micro-crystals on deeply patterned Si substrates. This growth method gives rise to GaAs epitaxial crystals, several micrometers wide, with no cracks and no threading dislocations^20,21^. However, the use of 3D growth mode is not convenient for actual device processing due to the micrometer size tiling of the surface intrinsic of this growth mode.

In addition to the above-mentioned issues, group IV materials (Si and Ge) are non-polar crystals. When III–V compound semiconductors, which are polar crystals, are grown on Si (or Ge) (001) substrates, formation of the AP domains may result^22,23^. Different AP domains are characterized by crystal orientations rotated of 90° along the growth direction. The domains are separated by the related AP boundaries, which may act as non-radiative recombination centers^24^. Monoatomic steps on the group IV substrate surface are the source of AP disorder in the compound side of the interface^25^. Consequently, it has been demonstrated that the nucleation of AP domains can be suppressed if the surface shows a majority of double-height atomic steps^24,26^. This step configuration can be achieved by using off-cut substrates and promoting step-bunching via annealing processes. Recently, the possibility to grow AP boundary free III–V semiconductors on Si and Ge has been demonstrated, through a careful tuning the surface step morphology^27–29^. Nevertheless, these techniques, even though very promising, cannot overcome the problem of wafer bowing and cracking for thick epilayers.

In this work we demonstrate a novel approach for the growth, directly on exact-(001) Si substrates, of high optical quality, large area epitaxial GaAs layers free of TDs, cracks and AP boundaries. Our approach exploits the uniform, suspended, Ge layer which can be obtained by merging, via thermal annealing cycles, of Ge 3D micro-crystals realized on deeply patterned Si substrates^30^. The suspended Ge layer acts as optimal virtual substrate for the GaAs growth, being closely lattice matched with GaAs, free of TDs, with a reduced thermal stress and no cracks^30–32^. The three-dimensional modulated (hilly) surface of this Ge suspended layer^30^, whose curvature can be controlled during the annealing process, is the key feature of our method to reduce the AP boundary issue in the GaAs. The characteristic continuous modulation of the surface curvature of this substrate provides the step density necessary to promote step bunching into the double-height steps configuration during annealing that is known to suppress the formation of AP domains in the GaAs epilayer. We also propose a three-temperature growth procedure to promote defect reduction and AP domain annihilation in the GaAs epilayer.

## Materials and Methods

**Figure 1:**
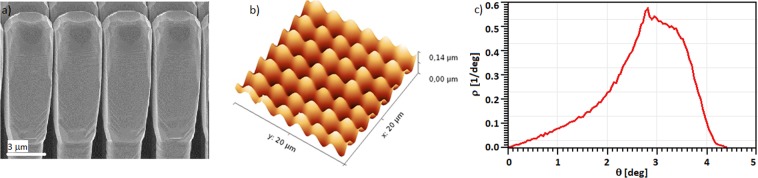
(**a**) SEM image of the Ge micro-crystals grown on Si pillars. (**b**) AFM topography of the Ge surface after the thermal annealing cycle. The surface shows a periodic undulation; the hills correspond to the position where the micro-crystals were before the merging. The valleys are the areas in between the micro-crystals. (**c**) probability density *ρ*(*θ*) to find an angle *θ* between the normal at the local sample surface and the [001] direction, extracted from the AFM image in (**b**). The distribution peaks at 2.8°, and 52% of the surface show an angle, respect to the (001) direction, higher than 3°.

The fabrication of the suspended Ge layer proceeds as follows. The growth of Ge 3D micro-crystals is carried out in a Low-Energy Plasma-Enhanced Chemical Vapor Deposition (LEPECVD)^33^ tool. A Ge layer, 8 μm thick, is grown on a deeply patterned nominal-(001) Si substrate. The substrate is patterned by 2 μm × 2 μm square pillars separated by 1 μm gap. The trenches between pillars are 8 μm deep. After the growth of 8 μm of Ge the surface is made of elongated micro-crystals^34^, separated by gaps, few tens of nanometers wide, perfectly tiling the substrate, as reported in Fig. 1a. Figure 1b shows the Ge surface after an *in-situ* thermal annealing cycle follows the growth, consisting in 6 cycles of 2 minutes each, formed by a ramp from 600 °C to 800 °C, which closes the gap between the micro-crystals and allows to obtain a suspended Ge layer^30^.

After the thermal annealing process, the wafer is moved into a Molecular Beam Epitaxy (MBE) equipment where the III-As growth is performed. The sample undergoes an annealing step at 680 °C, which has the double objective of cleaning the surface and enhance the formation of the double height atomic steps in Ge^24^. An As prelayer was then deposited at 580 °C before the growth.

**Figure 2:**
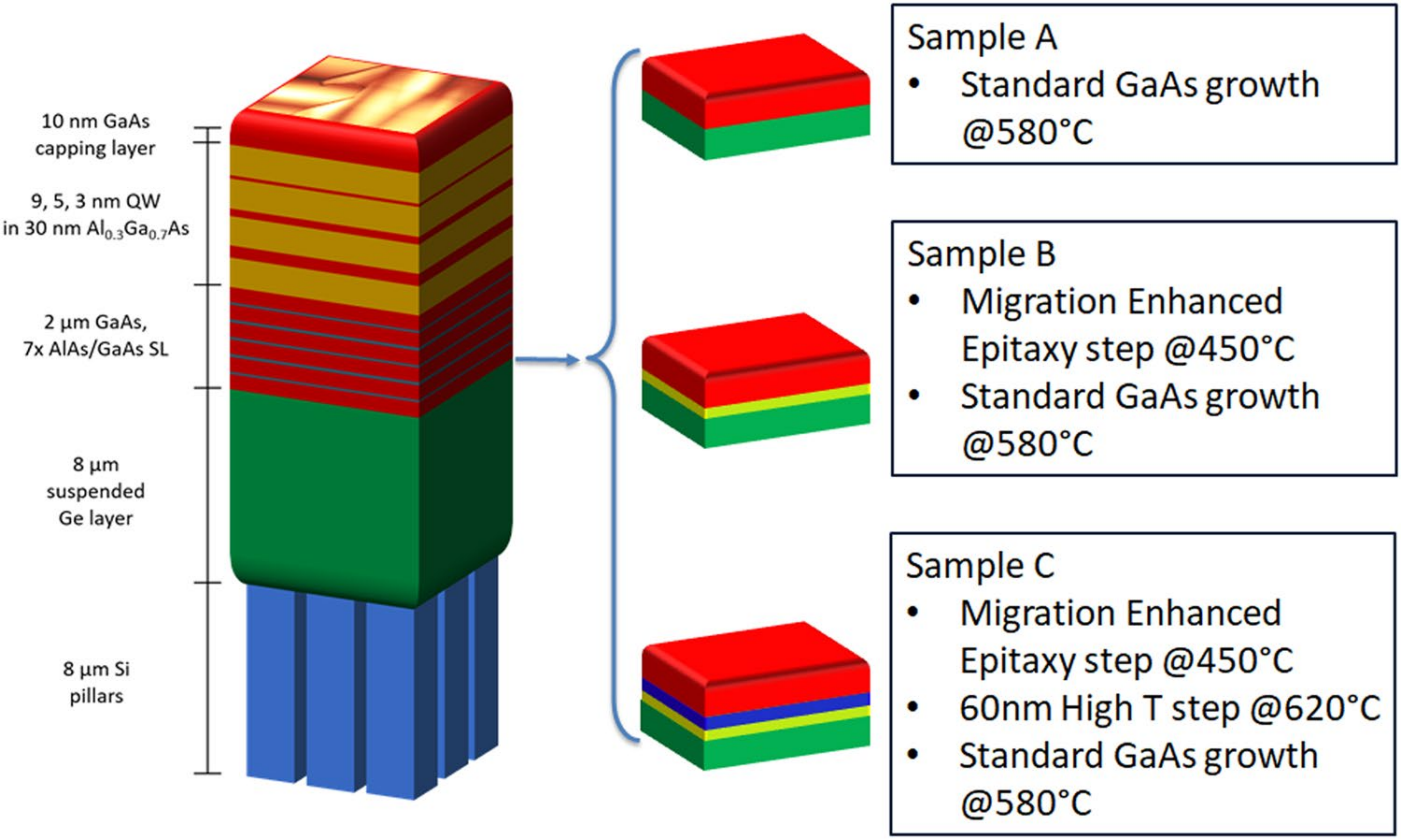
Schematic representation of the grown structures, highlighted on the right the three initial steps of the growth for the sample A, B and C, respectively.

Three different strategies were then used to initiate the growth of the GaAs epilayer (see Fig. 2). The first one (sample A) involves no changes in the standard growth procedure used to obtain high quality GaAs epilayers on Ge/Si (001) off-cut substrates^35,36^. The growth, performed at 580 °C with an As pressure of 5.5 × 10^−6^ mbar and a growth rate of 0.5 ML/s, starts with five periods of 5 monolayers (MLs) AlAs and 5 MLs GaAs superlattice (SL), followed by 250 nm of GaAs. The SLs are known to reduce the Ge migration in the GaAs layer^37,38^. This structure is then repeated 8 times reaching 2 μm height structure. On top of this layer, an optically active structure made by three quantum wells (QWs) of GaAs embedded in 30 nm Al_0.3_Ga_0.7_As barriers is realized. The width of the QWs is 9, 5 and 3 nm, from the deepest to the shallowest, respectively. Finally 10 nm of GaAs is grown as capping layer. In the second sample (Sample B), just after the initial As prelayer, a layer of 40 nm of GaAs grown by Migration Enhanced Epitaxy (MEE) at 450 °C is introduced. After this initial step, the temperature is raised to 580 °C and the same structure of Sample A is realized. In third sample (Sample C), the MEE layer is replicated and then is followed by 60 nm of GaAs grown by standard MBE at 620 °C. Then the usual 2 μm GaAs/SL structure plus the active region was grown as in Sample A. Figure 2 reports the schemes of the three samples A, B anc C.

Surface topography of the grown samples is measured by Atomic force microscopy (AFM) working in tapping mode and using a silicon tip capable of a lateral resolution of 20 nm. A conventional scanning electron microscope (SEM) is used to obtain images of grown structures.

A JEOL 3010 TEM operating at 300 kV is used for atomic resolution investigation. Transparent, cross sectional samples for TEM measurement are prepared using a standard procedure^39^. The sample is cut in two small pieces and embedded face-to-face into a special Ti-holder. The embedded samples are polished to an approximate thickness of 50 μm. Then the samples are placed into an ion mill for further thinning and are bombarded by 10 keV Ar^+^ ions, until the preparation of the TEM lamella. Ions in grazing angle are used for thinning to get a large transparent region around the perforation. The thinned specimen is bombarded further at 3 keV to decrease the ion beam damage at thinning.

The optical properties of the fabricated nanostructures are studied also by micro-photoluminescence (micro-PL). The sample is kept at 10 K in a low-vibration Janis ST-500 continuous He-flow cryostat which in turn is mounted on a translation stage for scanning the sample surface. The luminescence is collected by a home-made confocal microscope setup equipped with an infinity corrected 100x objective (Numerical Aperture = 0.7). The luminescence is spectrally dispersed and detected using an Acton SP2300i spectrograph mounting a 600 gr mm^−1^ grating and a 1200 gr mm^−1^ grating, blazed at 1000 nm and 750 nm respectively, and a Si CCD Acton Pixis 100F. The spatial resolution of the system is about 700 nm, while the spectral resolution is about 400 μeV using the 600 gr mm^−1^ grating and 250 μeV using the 1200 gr mm^−1^ grating. The excitation source is a CW diode-pumped solid-state laser at 532 nm.

## Results and Discussion

**Figure 3:**
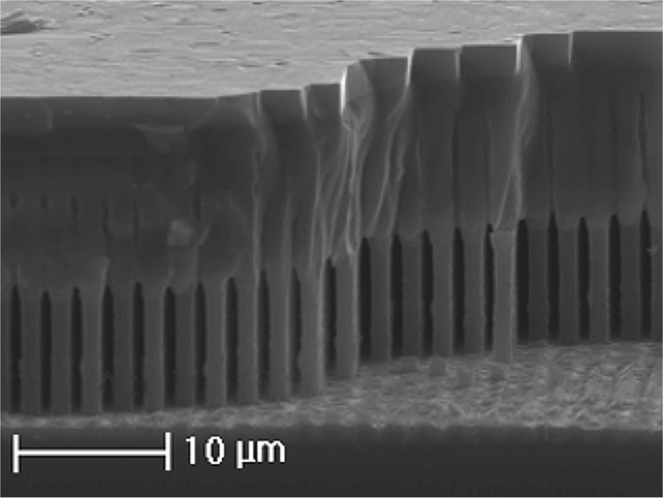
Cross-section SEM image of the whole structure of Sample A. It is possible to distinguish the separated Si pillars at the bottom, the merged Ge layer and the GaAs layer on the top.

An initial SEM analysis is carried out on the samples in order to check the uniformity of the Ge and GaAs layer. Figure 3 reports the morphology of the Ge suspended layer after the GaAs growth. The Ge layer presents voids between the pillars. Nonetheless it is characterized by a continuum top surface as the outcome of the annealing step. As expected the Si pillars at the bottom of the structure are not affected by the annealing and keep their original shape. The GaAs structure grown on the Ge suspended layer is a solid, uniform and continuum layer without voids or holes. The SEM measurements also show the absence of cracks in the Ge and GaAs layer, confirming that the thermal stress induced by the annealing and GaAs growth on the structure is released through the deformation of the Si pillars^31^.

The AFM of the suspended Ge layer shows the hilly surface, where the top of each hill marks position of each single Ge micro-crystal before the annealing step (see Fig. 1b). In order to describe the surface undulation, we report in Fig. 1c the probability density *ρ*(*θ*) to find an angle *θ* between the normal at the local sample surface and the [001] direction. In other words *ρ*(*θ*)*dθ* is the probability that the angle is in the interval (*θ*, *θ* + *dθ*) and obviously the ${\int }_{0}^{\pi /2}\,\rho (\theta )d\theta =1$. From this analysis we found that most of the surface is not parallel to the (001) direction, with more than 52% showing an angle, respect to the (001) plane, higher than 3°. The curvature, and hence the average angle, of the surface can be controlled via annealing temperature and time duration.

**Figure 4:**
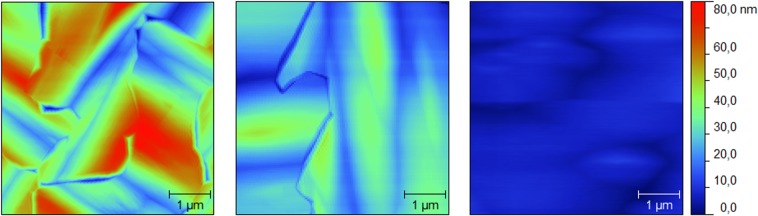
5 μm × 5 μmAFM topographies of (**a**) sample A, standard GaAs growth performed at 580 °C, (**b**) sample B with a preliminary Migration enhanced epitaxy (MEE) step at 450 °C then standard GaAs growth. (**c**) Sample C, MEE step at 450 °C, 50 nm high temperature MBE step at 620 °C, then standard GaAs growth.

From the AFM topography measurements of the GaAs epilayer (Fig. 4) it is possible to identify the presence of AP boundaries in the GaAs epilayer, as they appear as kinks at the surface^40^. This phenomenology arise from the instability of the AP boundaries caused by charge effects and strain accumulation, related to the presence of III–III or V–V bonds^41^.

Sample A (see Fig. 4a) shows a surface tessellated by AP domains with 3 μm average lateral size. The surface shows a relatively high roughness, with a root mean square (RMS) value of 15.3 nm. The observed 90° rotation in the growth anisotropy between adjacent domains is the outcome of the crystal rotation between AP domains. The AP domains are localized at the hill position, with the boundaries located in the negative curvature areas of the Ge surface, that is the valley between the hills. The overall AP domain distribution on the surface resembles a chessboard modulation with the domains usually covering completely one single hill and only sometimes extending to two or more hills. However, the occurrence of these expanded AP domains can be explained on the basis of pure stochastic phenomena, which leads two AP domains with the same orientation to lie on neighboring hills.

In order to increase the AP domain size, and thus in turn decrease the density of AP boundaries, we adopted for Sample B a two temperature strategy developed for the metalorganic chemical vapour deposition (MOVPE) of III–V on Si monolithic integration^42,43^. The procedure starts with a low temperature nucleation step performed with a high mobility growth mode (in our case a MEE step) followed by the growth of the epilayer at standard growth temperature. The initial step of the III–V growth on Si proceeds in the Volmer-Weber mode, thus characterized by separated islands that will coalesce during the growth. The high mobility, low temperature step, allows the nucleation of a high density of small islands that favors the self-annihilation of the AP domains within few nanometers from the interface and generates a lower density of disorder defects like stacking faults and twins^42^. The surface morphology of Sample B shows a reduction in the surface roughness (RMS = 8.1 nm) and a loss of the correlation between the GaAs and the substrate morphology. Domains dimension is larger than in Sample A, reaching an average size of 5 μm. This indicates the effectiveness of the low temperature, high mobility step, before the growth. Still, the observed density of AP boundaries in Sample B is far from optimal for opto-electronic integration on Si.

According to ref.^42^, when the III–V epilayer is subjected to high temperature, the AP boundary lying in {110} plane breaks-up in two boundaries, laying on {111} planes. AP boundaries propagating in these directions have a higher probability to self-annihilate thus reducing the presence of AP domains at the surface. However, MOVPE growth temperature is higher than in MBE. Thus a simply imitative procedure, recovering the usual MBE after the low-temperature step, could reach a temperature not high enough to produce the expected decomposition of {110} AP boundaries. For this reason, a three-temperature procedure is introduced in Sample C. Following the MEE step for the nucleation of small island, a high temperature growth step is performed, where 60 nm of GaAs are grown at 620 °C^43^ to promote AP boundaries dissociation.

Sample C, as expected (Fig. 4c), presents a single AP domain, covering all the 5 μm × 5 μm area showed in the picture. The surface roughness is quite small (RMS= 2.2 nm). On average, the lateral size of AP domains in Sample C is 8 μm. Nonetheless, large area domains are present on the surface and it was possible to find domains with a lateral dimension reaching 30 μm.

**Figure 5:**
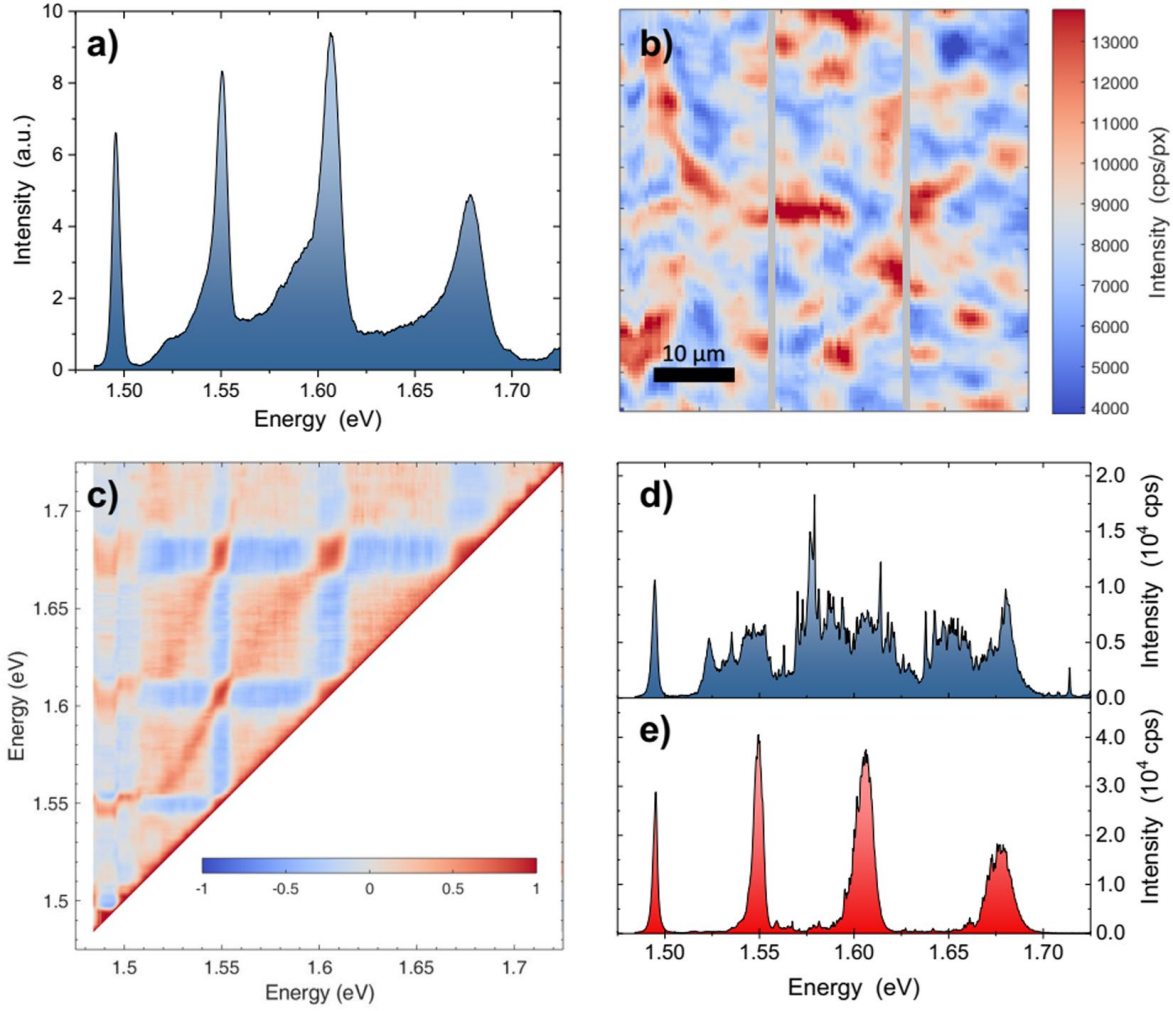
PL analysis of a hyperspectral map of 50 μm × 50 μm, step 0.5 μm performed on sample C. (**a**) Macro-PL obtained summing up all the spectra of the hyperspectral map. (**b**) Intensity map of the emission of QW1 (1.550 eV) obtained integrating the hyperspectral map between 1.537 and 1.556 eV. (**c**) Pearson correlation analysis of the hyperspectral map. (**d**,**e**) Two spectra selected from the hyperspectral map in (**b**), representative of low and high signal regions, respectively.

Micro-PL hyperspectral map is used to evaluate the crystalline and optical quality of Sample C. The map is acquired over a 50 μm × 50 μm region with an excitation power of 2 μW (about 200 Wcm^−2^). Figure 5a reports the PL spectrum of sample C obtained by averaging the spectra over the sampled region. Four peaks are clearly identified: 1.495 eV, 1.550 eV, 1.605 eV, 1.680 eV. The last three peaks are attributed to the QWs signals, from the 9, 5 and 3 nm QW, respectively. The peak at 1.495 eV is attributed to conduction band to neutral carbon acceptor recombination (e, C) since this emission matches the value reported in the literature and since a small carbon contamination is always present^44^. Regarding the presence of Ge related emissions in our PL spectra, it can be reasonably excluded, because no Ge diffusion is expected in the QW region due to the superlattice blocking layer (see Materials and Methods section). Moreover the Ge related emissions in GaAs^44^, and in AlGaAs^44,45^ are all outside the energy range covered by our PL spectra.

The QW emission bands turn out to be spectrally broad, with a FWHM of about 8, 12 and 15 meV for the 9, 5 and 3 nm QW, respectively. Such a broadening can be ascribed to the non-planar geometry of the QWs on top of the pillars. Using a simple effective mass model to estimate the QW width fluctuations ΔL from the PL FWHMs, we obtain ΔL equals to 0.65, 0.25, and 0.12 nm for the 9, 5 and 3 nm QW, respectively. The QWs width fluctuations is reduced, going from the deepest to the shallowest. Moreover, the QW peaks show a clear asymmetric shape with a tail towards lower energies. This is clearly due to defect levels near the band edges, due to disorder.

In Fig. 5b we report the intensity map of the 9 nm QW obtained by integrating the hyperspectral map between 1.537 eV and 1.556 eV. Each pixel has a dimension of 0.5 μm × 0.5 μm. Intensity variations on the scale of several micrometers are clearly observed. This characteristic length can be related to the AP domains of the material. The maps for the other QWs are similar to the one in Fig. 5b. The correlation between the QW peaks can be investigated by a Pearson correlation analysis^46^. The result is reported in Fig. 5c where a positive correlation exists between the QWs signal. If a QW has a sharp and intense peak, so do the other peaks. It is also interesting to note that the three QW peaks are fairly correlated with the emission at 1.495 eV. In particular, the broader the peak at 1.495 eV, the larger the asymmetry of the QW.

The Fig. 5d,e show the spectra of two point selected from the map reported in Fig. 5b, in correspondence of a low and high signal region, respectively. The peak at 1.495 eV is visible in both spectra since the signal comes from the lower GaAs layer. The signals from the QWs are clearly visible in the high emission regions only (Fig. 5e), while in low emission regions no clear QW signal is present (Fig. 5d).

The reduction of the PL emission is attributed to the presence of AP boundaries. When the carriers are within the capture area of the AP boundary, the emission from QW is suppressed, not showing any clear peaks (Fig. 5d). Only far from the boundaries it is possible to observe the PL peaks of the QWs. Actually, from the performed micro-PL spatial measurements, there is no one-to-one correlation between the AP domains size at the surface and high/low emission regions. However, this is not surprising, since the link between the QWs properties and the presence of defects for hundreds of nm below the surface is quite complex to model out. We guess that the QW emission is mainly affected by the presence of AP boundaries which do not propagate to the surface.

**Figure 6:**
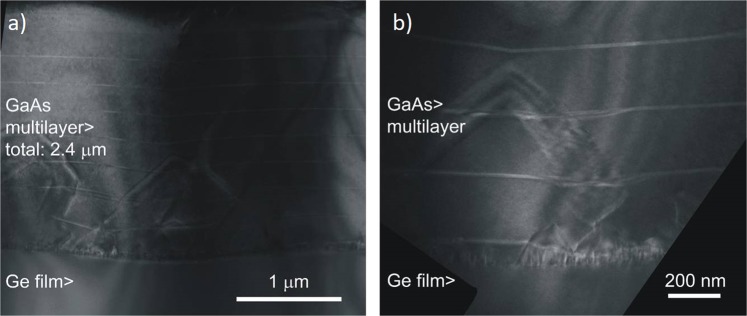
TEM micrographs of two regions of the sample C, with (**a**) a low magnification and (**b**) a high magnification. In the picture self-annihilation is visible. The bright longitudinal lines are the AlAS/GaAs SLs introduced every 250 nm.

The tentative interpretation we have proposed in Sample C is supported by the actual sample structure as observed by cross-sectional TEM analysis (see Fig. 6). As expected on the basis of AFM measurements, the TEM images show that no AP boundary propagates up to the sample surface. The majority of AP domains annihilate within the initial 100 nm from the interface, which correspond to the low/high temperature initial growth steps. Some AP boundaries propagate, following {111} planes, up to 1 μm inside the GaAs epilayer where they eventually annihilate. No stacking faults or dislocations are observed. The TEM analysis thus confirms the effectiveness of the combination of hilly surface and three-temperature approach to reduce defects and promote AP boundaries removal by enhancing self-annihilation.

The presence of AP boundaries propagating well within the GaAs layer agrees with our explanation of the observed modulation of the micro-PL intensity and spectral characteristics. In particular, the growth front evolution with time can be measured through the profile of the AlAs/GaAs SLs, which can be easily spot in the TEM images as equally spaced bright lines. In the areas far apart from AP boundaries, the surface shows a progressive smoothing driving the system towards a flat epilayer. Conversely, in the area close to the AP boundaries that propagate deeply into the GaAs layer, the TEM image shows a defected and rough growth front, which causes intermixing and defects. This observation agrees with the observed correlation between QW spectral broadening, intensity reduction and increase of impurity incorporation. All these effects are, in fact, present in the material volumes close to the AP boundaries.

## Conclusions

Defect-free GaAs growth on exact (001) Si substrates is an important prerequisite for integrating III/V-based device layers with Si-based micro-electronics. In our approach we used two fundamental innovations that permit the achievement of large area epitaxial GaAs layers free of TDs, cracks and AP boundaries, with high optical quality. The first innovation is the use of a suspended Ge layer which can be fabricated on deeply patterned Si substrates^30^. This Ge layer is free from TD and lattice matched with the GaAs. It can efficiently relax the thermal strain which can arise between the epilayer and Si substrate as a consequence of the growth procedure, thus avoiding the formation of cracks^31^. In addition, the three-dimensional modulated (hilly) surface of the suspended layer, owing to its intrinsic curvature, permits the formation, via annealing induced step bunching, of double-height steps on the Ge surface. Although capable to reduce the density of AP boundaries, the sole use of the substrate curvature is not enough. For this reason we developed an innovative three-temperature growth process, to maintain the initial growth islands small and promote AP boundary annihilation. This combination results in a GaAs epilayer characterized by large AP domains, with a lateral size of up to 30 μm, grown on Si (001) substrate.
